# Biochemical Characterization of *Kluyveromyces lactis* Adenine Deaminase and Guanine Deaminase and Their Potential Application in Lowering Purine Content in Beer

**DOI:** 10.3389/fbioe.2018.00180

**Published:** 2018-11-29

**Authors:** Durga Mahor, Gandham S. Prasad

**Affiliations:** Microbial Type Culture Collection and Gene Bank, CSIR-Institute of Microbial Technology, Chandigarh, India

**Keywords:** *Kluyveromyces lactis* (*K. lactis*), hyperuricemia, gout, purine, high-performance liquid chromatography (HPLC), beer

## Abstract

Excess amounts of uric acid in humans leads to hyperuricemia, which is a biochemical precursor of gout and is also associated with various other disorders. Gout is termed as crystallization of uric acid, predominantly within joints. The burden of hyperuricemia and gout has increased worldwide due to lifestyle changes, obesity, and consumption of purine-rich foods, fructose-containing drinks, and alcoholic beverages. Some of the therapies available to cure gout are associated with unwanted side-effects and antigenicity. We propose an attractive and safe strategy to reduce purine content in beverages using enzymatic application of purine degrading enzymes such as adenine deaminase (ADA) and guanine deaminase (GDA) that convert adenine and guanine into hypoxanthine and xanthine, respectively. We cloned, expressed, purified, and biochemically characterized both adenine deaminase (ADA) and guanine deaminase (GDA) enzymes that play important roles in the purine degradation pathway of *Kluyveromyces lactis*, and demonstrate their application in lowering purine content in a beverage. The popular beverage beer has been selected as an experimental sample as it confers higher risks of hyperuricemia and gout. Quantification of purine content in 16 different beers from the Indian market showed varying concentrations of different purines. Enzymatic treatment of beer samples with ADA and GDA showed a reduction of adenine and guanine content, respectively. These enzymes in combination with other purine degrading enzymes showed marked reduction in purine content in beer samples. Both enzymes can work at 5.0–8.0 pH range and retain >50% activity at 40°C, making them good candidates for industrial applications.

## Introduction

Hyperuricemia, an elevated level of uric acid, is an independent risk factor for hypertension, metabolic, and cardiovascular disorders. It also causes painful arthritis known as gout (Chen et al., 2007; Kuwabara, 2016) and it is the most common inflammatory arthritis, affecting 1–2% of the adult population (Richette and Bardin, 2010). Hyperuricemia and gout both occur due to elevated levels of uric acid in the human body. Low urate excretion and higher production of urate, or both conditions, lead to hyperuricemia. Various reports also suggest that epidemiologically, the prevalence of gout and hyperuricemia has increased worldwide, especially in developed countries (Kuo et al., 2015). Numerous factors (modifiable and non-modifiable) like sex, age, race, alcohol consumption, obesity, and lifestyle change regulate hyperuricemia and gout (Choi et al., 2005; Choi and Curhan, 2008; MacFarlane and Kim, 2014). Among these factors, some are modifiable (lifestyle change and alcohol consumption) and their regulation can reduce the prevalence of hyperuricemia and gout (MacFarlane and Kim, 2014). Although many therapies and medications are available for gout treatment, cost-effectiveness and side effects are factors to consider (Cronstein and Terkeltaub, 2006). Despite various medication therapies' availability, a low purine diet and a healthy lifestyle are recommended for all gouty patients (Choi and Curhan, 2004; Choi et al., 2004b). A low purine diet and lifestyle change are considered as some of the alternatives and safe approaches and have the potential to regulate the prevalence of gout and hyperuricemia. The low purine diet recommendation is promising and safe, but it eliminates a wide range of foods and beverages, which makes therapy difficult to be adopted by a major percentage of patients. One of the strategies to make this therapy safe and attractive is to produce the foods and beverages that are low in purine content through the enzymatic application (Trautwein-Schult et al., 2014; Jankowska et al., 2015; Mahor et al., 2016). This gives the patients an option to choose a wide variety of low purine content foods and beverages without any risk of hyperuricemia and gout.

We employed this strategy to produce low purine content beverages through the application of purine degrading enzymes of a GRAS status yeast *Kluyveromyces lactis*. Our group is working on the enzymes involved in the purine degradation pathway of *K. lactis*, an industrially important and Food and Drug Administration (FDA)-approved generally regarded as safe (GRAS) status yeast. We have earlier worked on uricase and purine nucleoside phosphorylase of this organism (Kumar, 2012; Mahor et al., 2016). In the present study, we mainly focused on adenine deaminase and guanine deaminase enzymes of the purine degradation pathway of *K. lactis* and their application in lowering purine content in beverages.

Adenine deaminase (ADA) (EC 3.5.4.2.) is also known as adenine amidohydrolase and adenase. It is an intracellular enzyme that deaminates adenine into hypoxanthine with the release of ammonia. ADA is conserved in prokaryotes and lower eukaryotes (Pospísilová et al., 2008). ADA has been reported from *Pseudomonas synxantha* (Jun and Sakai, 1985), *Bacillus subtilis* (Nygaard et al., 1996), *Leishmania donovani* (Kidder and Nolan, 1979), *E. coli* (Matsui et al., 2001), *Azotobacter vinelandii* (Heppel et al., 1957), and *Arxula adeninivorans* (Jankowska et al., 2014). It belongs to the α/β barrel subfamily and most ADAs require metal ions for their catalysis, e.g., *P. synxantha* and *B. subtilis* (Jun and Sakai, 1985; Nygaard et al., 1996).

Guanine deaminase (GDA) (EC 3.5.4.3) is a key enzyme of the purine catabolism that belongs to the aminohydrolase family (Schmidt, 1932). It is commonly known as cypin, nedasin, guanase, aminase, guanine aminohydrolase, and guanine aminase. GDA catalyzes guanine and produces xanthine and ammonia via hydrolytic deamination (Lewis and Glantz, 1974; Fernández et al., 2009). It is an irreversible reaction and guanine is reutilized for guanylate nucleotide pools (Fernández et al., 2009). GDA is found in bacteria, lower eukaryotes, plants, and higher eukaryotes (Liaw et al., 2004). GDA has been categorized into two different classes: the aminohydrolases family found in bacterial, fungal, and many eukaryotes and the cytidine deaminase family found in plants and archaea (Ko et al., 2003; Liaw et al., 2004). A number of tissues have not shown a steady level of GDA expression (Scholar and Calabresi, 1973; Kuzmits et al., 1980). Tissue-specific GDA expression suggested that it plays an important role in guanine nucleotide pools (Berger et al., 1985). GDA also has a potential role in cofactor and nucleic acid precursor and signaling pathways. It is a promising drug target for liver and cognitive diseases (Fernández et al., 2010). Similarly, other purine-degrading enzymes (purine nucleoside phosphorylase, xanthine oxidase, and uricase) also play important roles in various disorders (Bzowska et al., 2000; Glantzounis et al., 2005). However, there are very few studies that report ADA and GDA homologs from yeast.

So far, of purine-degrading enzymes, only single yeast *A. adeninivorans* are characterized (Jankowska et al., 2013, 2015; Trautwein-Schult et al., 2013, 2014). In the present study, we report the biochemical characterization of *Klac*ADA and *Klac*GDA and their application in lowering purine content in beer. Numerous studies reported that alcohol consumption is strongly associated with gout and the risk of gout attack conceivably depends on the type and dose of alcohol (Choi and Curhan, 2004, 2008; Choi et al., 2004a). The presence of absorbable nucleosides in beer confers a greater risk of gout in comparison to any other alcohol beverages (i.e., wine and spirits).

Previously, we have overexpressed and purified purine nucleoside phosphorylase (Mahor et al., 2016) and xanthine dioxygenase (Mahor et al., 2016, unpublished data) enzymes from *K. lactis*. Using all these enzymes, we demonstrated that applying an enzyme cocktail (*Klac*ADA, *Klac*GDA, *Klac*PNP, and *Klac*XanA) is able to reduce purines viz. adenine, guanine, inosine, and xanthine content in beer. The enzyme cocktail, along with commercial uricase, was able to reduce overall purine content in beer. The characterization and application of these enzymes support for a method development for low purine content food production.

## Materials and methods

### Cloning, overexpression, and purification of *Klac*ADA and *Klac*GDA

Genomic DNA of *K. lactis* (MTCC 458) was isolated using a Zymo DNA isolation kit (Zymo Research, USA). Amplified products of *Klac*ADA and *Klac*GDA were cloned into a pET28c vector with N-terminal 6xHis-tag by using NdeI/XhoI restriction sites. Clones pET28c-ADA-DH10β and pET28c-GDA-DH10β were overexpressed in *E. coli* BL21(DE3) cell and followed by induction with Isopropyl β-D-1-thiogalactopyranoside (IPTG). Positive clones were confirmed by gene-specific PCR amplification, digestion, and followed by sequencing. Best overexpressed clones of *Klac*ADA and *Klac*GDA were inoculated into 10 mL of primary culture in LB + 50 μg/mL of kanamycin. One percent of primary inoculums were inoculated into 1 L of LB + kanamycin media for a large-scale culture incubated at 37°C, induced with IPTG and incubated at 30 and 25°C, respectively, for 7 and 12 h. Cells were harvested by centrifugation at 4,279 × *g* for 15 min at 4°C. Pellets of *Klac*ADA and *Klac*GDA were lysed with lysis buffer (50 mM Tris, 150 mM NaCl, 10 mM imidazole pH 7.0 and 50 mM Tris, 200 mM NaCl, 10 mM imidazole, pH 8.0, respectively), along with protease cocktail inhibitor (1 μL/mL, Sigma- Aldrich), lysozyme 1 mg/mL followed by 30 min incubation. Disruption of cells was done by sonication for 1 h and cell debris separation done by centrifugation at 18,400 × *g* at 4°C for 45 min. The supernatant of both proteins was loaded into the Ni-NTA column which was pre-equilibrated with buffers. Ni-NTA bound proteins were eluted in several fractions in 50 mM Tris, 150 mM NaCl, 200 mM imidazole, 15% glycerol, pH 7.0 and 50 mM Tris, 200 mM NaCl, 200 mM imidazole, and 10% glycerol pH 8.0, respectively. All fractions of purified proteins were pooled and *Klac*ADA and *Klac*GDA were dialyzed against 25 mM Tris, 100 mM NaCl, 1 mM DTT, 15% glycerol, pH 7.0 and 50 mM Tris, 100 mM NaCl, 10% glycerol, and pH 8.0 overnight at 4°C. Determining the purified protein concentration was done by UV spectra at 280 nm.

### Oligomerization of *Klac*ADA and *Klac*GDA

The oligomeric state of *Klac*ADA and *Klac*GDA was determined by gel filtration chromatography. Dialysed recombinant proteins were concentrated and *Klac*ADA and *Klac*GDA were further purified by gel exclusion chromatography by using HiPrep 16/60 Sephacryl S-200 HR column and HiPrep 16/60 Sephacryl S-300 HR column (GE Healthcare Life Sciences), respectively. Further confirmation of results was done by MALDI-TOF analysis. MALDI mass spectra of *Klac*ADA and *Klac*GDA was generated by using sinapinic acid as a matrix on a mass spectrometer (Agilent Technologies G4226A, US).

### Enzymatic assays of *Klac*ADA and *Klac*GDA

ADA converts adenine into hypoxanthine with the release of ammonia and *Klac*ADA activity was measured spectrophotometrically monitoring adenine consumption at 254 nm. Enzyme assay was done at 30°C and in 100 mM potassium phosphate buffer pH 6.0. The reaction consisted 200 nmoles of the enzyme, 100 mM of the buffer in 250 μL of the final volume and was incubated for 20 min. The adenine concentration varied from 0 to 1 mM to calculate kinetic parameters. In addition to the above-mentioned assay, we have also determined *Klac*ADA activity through an indirect assay at 30°C under the following assay conditions: 0.1 U xanthine oxidase, 100 mM potassium phosphate buffer pH 6.0, 200 nmoles of enzyme, and 0–1 mM of adenine. Uric acid production was monitored in the assay at 293 nm.

Guanine was converted into xanthine by guanine deaminase. Guanine deaminase activity was determined by monitoring guanine utilization at 250 nm. The assay reaction consisted of 0.5 μM of the enzyme, 100 mM phosphate buffer pH 7.0, and was incubated for 15 min at 25°C. The guanine concentration varied from 0 to 500 μmoles and was stopped at 10, 20, and 30 min to get the linear range. The kinetic parameters of *Klac*GDA were calculated by non-linear parameters of origin software. *Klac*GDA activity was also determined by indirect assay by using 0.1U xanthine oxidase in 100 mM phosphate buffer pH 7.0, and 0.5 μM of the enzyme at 25°C. The absorbance was monitored at 293 nm for uric acid production.

### Biochemical properties of *Klac*ADA and *Klac*GDA

The effect of pH on *Klac*ADA and *Klac*GDA activity was determined by their measured activity in 100 mM universal buffer (50 mM Tris, 50 mM boric acid, 33 mM citric acid, and 50 mM Na_2_PO_4_) (Visser et al., 2010) in a pH range of 3.0–12.0, at 1.0 pH unit interval. Reactions were stopped by the addition of 50 μL of 10 M NaOH and their activity was measured at the 254 and 250 nm, respectively. The optimum temperature of *Klac*ADA and *Klac*GDA was examined by measuring their activity across a 5–55°C temperature range. The *Klac*ADA and *Klac*GDA reaction mixtures in 100 mM universal buffer at optimum 6.0 and 7.0 pH levels, respectively, were incubated at 5–55°C with 5°C intervals. The reactions were stopped by adding 50 μL of 10 M NaOH, and absorbance was measured.

To determine the pH stability of *Klac*ADA and *Klac*GDA, proteins were incubated in a pH range of 3.0–12.0, at 1.0 pH unit interval of universal buffer for 12 h. The enzyme assay was performed under optimum conditions (*Klac*ADA at 25°C, pH 6.0 and *Klac*GDA at 30°C, pH 7.0) with incubated enzymes and reactions were stopped by 10 M NaOH. Finally, activity was measured at 254 and 250 nm, respectively. The thermostability of *Klac*ADA and *Klac*GDA were measured by incubating enzymes at 5–55°C for 1–2 h. Enzyme reactions were set with incubated enzymes at their optimum conditions and absorbance was measured at their respective wavelengths.

### Substrate specificity of *Klac*ADA and *Klac*GDA

Substrate specificity of enzymes was measured through the HPLC method. Standards of the substrate and expected products in 2 mM concentration were run on an SCL-10AV HPLC system (Shimadzu, Japan), equipped with a SIL-10AD injector and a UV spectrophotometric detector. Each of the substrates was treated with *Klac*ADA and *Klac*GDA in optimum conditions and incubated for 30 min. Post incubation, reaction mixtures were heated to 100°C to stop the reaction, centrifuged for 15 min and the supernatant was separated. Ten microliters of the sample was injected into in a Lichrospher 100 RP-18 column (LichroCART® 250-4, Merck), at 0.5 mL/min run at 35°C, and UV-absorbance spectra were collected at 254 nm. The solvent system used for separation was 100 mM sodium phosphate buffer + 10% methanol. The buffer pH was 2.3, maintained by 100 mM phosphoric acid. Chromatograms were analyzed in the absence and presence of enzymes which revealed that *Klac*ADA and *Klac*GDA can consume a wide range of substrates.

### Purine content quantification in beers

Pure purine compounds (adenosine, guanosine, inosine, xanthosine, adenine, guanine, hypoxanthine, xanthine, and uric acid) in a wide range of concentrations (0–5 mM) were run on the HPLC system to generate a standard curve, using the parameters mentioned earlier. Different beer samples designed with code words were run on HPLC without any pre-treatment. Analysis of HPLC chromatograms revealed the presence of different purines. The identity of each purine was determined by the retention times obtained for each individual pure purine compound chromatogram and was further confirmed by LC-ESI-MS analysis.

### Reduction of purine content in beer by enzymatic reaction

Standards (adenine, hypoxanthine, guanine, and xanthine) in 2 mM concentrations were dissolved in a phosphate buffer and were run on the HPLC system. HPLC parameters were the same as mentioned previously. To examine enzyme potential for purine reduction initially, the pure substrate was treated with *Klac*ADA and *Klac*GDA in 1.0 U at room temperature. HPLC chromatograms showed a decrease in adenine and guanine peaks and a corresponding increase in hypoxanthine and xanthine peaks. Beer samples treated with *Klac*ADA and *Klac*GDA (1.0 U) at room temperature were run on the HPLC system. Chromatograms of enzymatically treated and untreated beer samples revealed that both *Klac*ADA and *Klac*GDA were able to lower adenine and guanine content in beer. Retention time of standards was used to determine the identity of the compounds and peak value was used for quantification, and further confirmation was done by LC-ESI-MS analysis.

Finally, beer was treated with an enzyme cocktail (*Klac*ADA, *Klac*GDA, *Klac*PNP, *Klac*XanA in 1.0 U) along with uricase 0.2 U (commercially available as *Candida utilis* uricase), incubated for 1.5 h, and the sample was run on HPLC by using similar parameters as mentioned before. The enzymatic cocktail lowered the purine content in beer. The identification of purine compounds was done in a similar manner as mentioned above.

## Results

### Identification of *Klac*ADA and *Klac*GDA

The gene coded for ADA in *K. lactis* (gene ID KLLAOE2317) was found by a BLAST search with other available sequences. It is located from position 2,064,516–2,065,583 on the E-chromosome of *K. lactis* with no introns. The *Klac*ADA sequence showed a high degree of similarity with the ADA of *Kluyveromyces marxianus* (76%) and AAH of *S. cerevisiae* (66%), whereas the human, *E. coli*, bovine, and murine ADAs showed a 24, 28, 24, and 24% degree of similarity, respectively (Figure S1). This similarity goes well with the statement that lower eukaryotes have adenine deaminase rather than adenosine deaminase. The gene coding for GDA (gene ID. KLLAOD9492), located on chromosome D from position 1,643,703–1,645,193, was found by a BLAST search with fungal and higher eukaryotes GDAs sequences. Sequence similarity results revealed that *Klac*GDA shares a 69% sequence similarity with *K. marxianus* GDA, whereas S*. cerevisiae* GDA (51%), human GDA (37%), and *E. coli* GDA (35%) have less sequence similarity (Figure S2).

### Cloning, expression, and purification of *Klac*ADA and *Klac*GDA

The genomic DNA of *K. lactis* MTCC 458 was isolated by using a DNA isolation Kit (Zymo Research, USA). Amplified *ada* and *gda* genes were cloned into a pET28c vector with a NdeI/XhoI site carrying N-terminal 6xHis-tag. To overexpress enzymes, genes were further cloned into BL21(DE3) cells and induced with 0.300 and 0.150 mM of IPTG, respectively. Overexpressed proteins *Klac*ADA and *Klac*GDA were purified through a Ni-NTA column in several fractions. Purified fractions of proteins were pooled and further purified by gel filtration chromatography. SDS-PAGE results indicated that proteins were purified to >95% homogeneity. The *Klac*ADA can be stored at 4°C in 25 mM Tris, 100 mM NaCl, 15% glycerol and pH 6.0, and *Klac*GDA can be stored at 4°C in 50 mM Tris, 100 mM NaCl, 10% glycerol and pH 7.0, respectively, for a month.

### Oligomeric states of *Klac*ADA and *Klac*GDA

**Figure 1:**
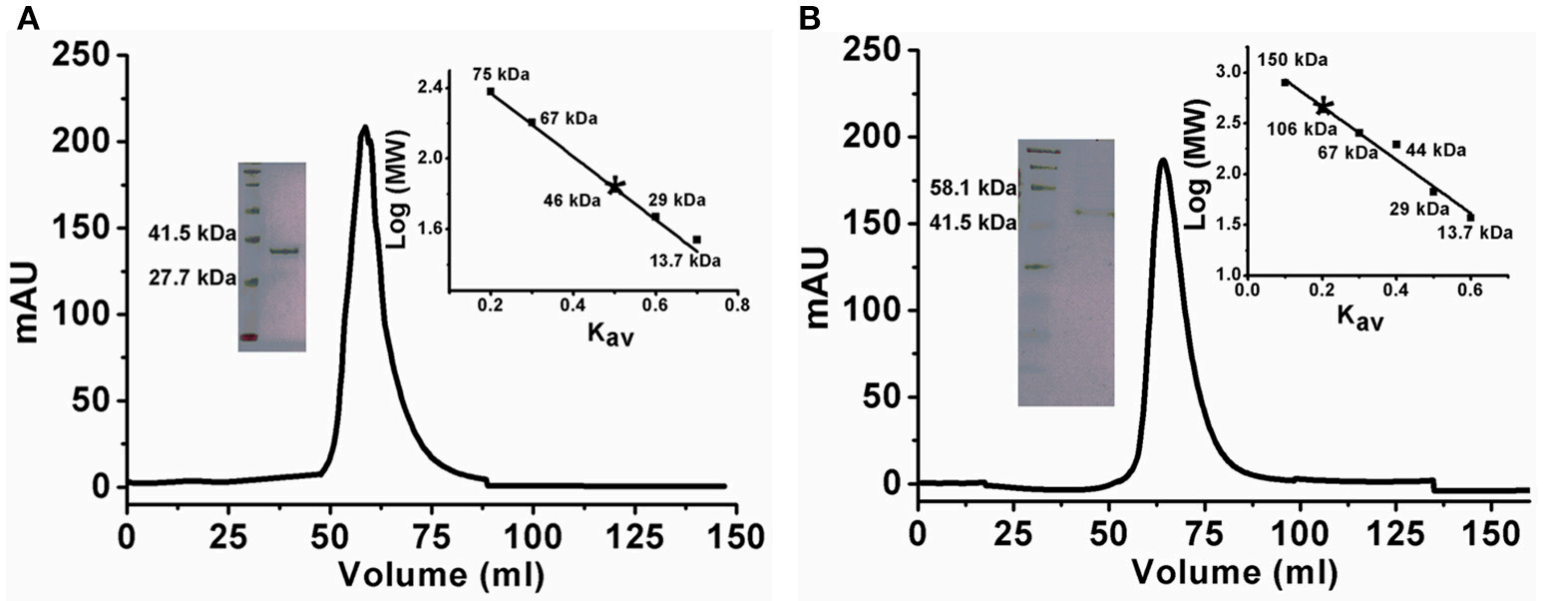
Gel exclusion chromatography elution profiles of *Klac*ADA and *Klac*GDA. **(A)** *Klac*ADA in HiPrep 16/60 Sephacryl S-200 HR column. The inset graph shows the elution profile of standard molecular weights from the same column ribonuclease (13.7 kDa), carbonic anhydrase (29 kDa), BSA (67 kDa), conalbumin (75 kDa), (left panel) and SDS-PAGE profile of *Klac*ADA with protein ladder (right panel). **(B)** *Klac*GDA in HiPrep 16/60 Sephacryl S-300 HR column. The inset graph shows the elution profile of standard molecular weights from the same column ribonuclease (13.7 kDa), carbonic anhydrase (29 kDa) ovalbumin (44 kDa), conalbumin (75 kDa), alcohol dehydrogenase (150 kDa) (left panel) and SDS-PAGE profile of *Klac*GDA with protein ladder (right panel).

There are a few reports of an oligomeric form of ADA (Jankowska et al., 2015). To confirm the oligomeric state of *Klac*ADA, we performed gel exclusion chromatography. A single dominant peak at 60 mL, which corresponds to 46 kDa molecular weight, indicates a monomeric form of *Klac*ADA (Figure 1A), while a single peak of *Klac*GDA corresponding to 106 kDa suggests a dimeric form of it (Figure 1B). These results were very well-correlated with MALDI-TOF analysis (Figure S3).

### Biochemical properties of *Klac*ADA and *Klac*GDA

**Figure 2:**
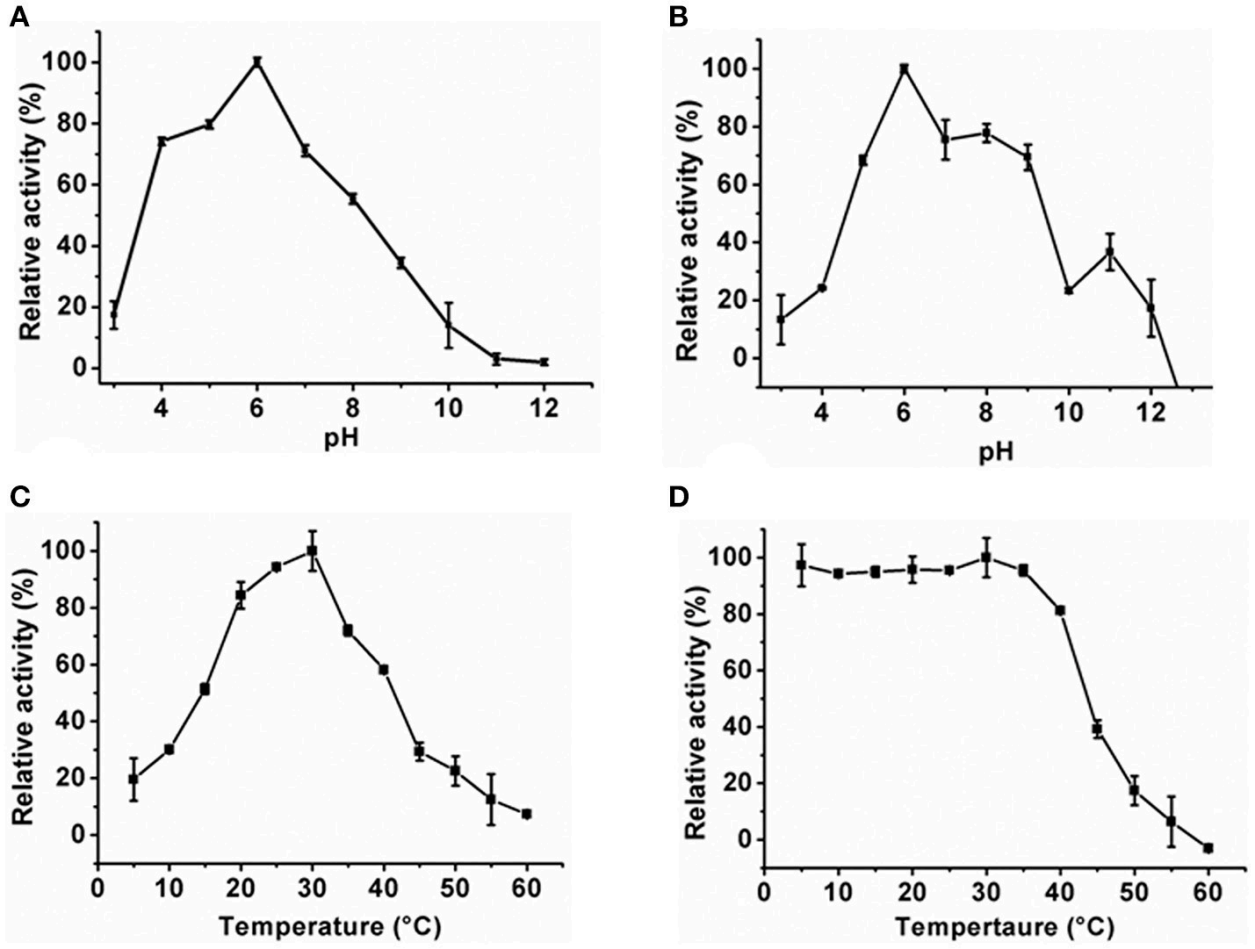
Biochemical properties of *Klac*ADA. **(A)** Optimum pH of *Klac*ADA. **(B)** Effect of pH on *Klac*ADA stability. **(C,D)** Optimum temperature and thermostability of *Klac*ADA. The experiments were done in triplicates and representing values are means of them. The activity of enzymes measured in optimum conditions and maximum activity was defined as having a relative activity of 100%. Standard deviation represented by error bars.

**Figure 3:**
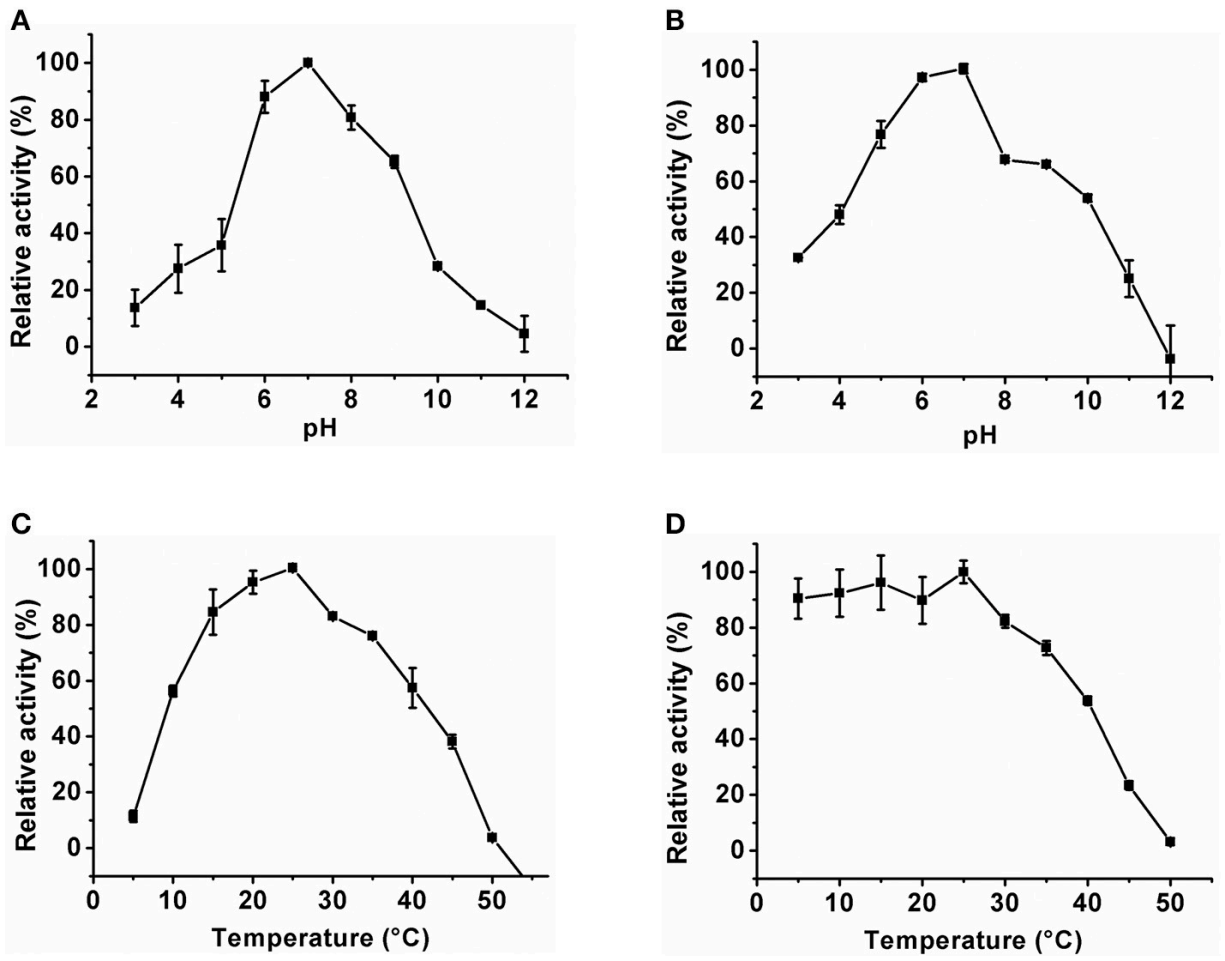
Biochemical properties of *Klac*GDA. **(A)** Optimum pH of *Klac*GDA. **(B)** pH stability of *Klac*GDA. **(C,D)** Optimum temperature and thermostability of *Klac*GDA. The experiments were done in triplicates and representing values are means of them. The activity of enzymes measured in optimum conditions and maximum activity was defined as having a relative activity of 100%. Standard deviation represented by error bars.

The optimum pH of the *Klac*ADA and *Klac*GDA was determined using a universal buffer with different pH ranges. It was observed that *Klac*ADA is able to work at a pH range of 4.0–8.0 with an optimum pH of 6.0 (Figure 2A), and *Klac*GDA can work at pH 6.0–9.0 with an optimum pH of 7.0 (Figure 3A). *Klac*ADA and *Klac*GDA are able to work across a wide temperature range with an optimum temperature of 30°C (Figure 2C) and 25°C (Figure 3C), respectively. *Klac*ADA worked in pH range of 4.0–8.0 with >50% activity (Figure 2B), whereas *Klac*GDA retained >40% catalytic activity at a pH range of 5.0–9.0 (Figure 3B). The enzymes were moderately thermostable in nature; *Klac*ADA retained >85% activity after incubation at 5–40°C (Figure 2D), while *Klac*GDA retained >50% activity after incubation at the same range of temperature for 1.5 h (Figure 3D).

### Kinetic analysis of *Klac*ADA and *Klac*GDA

Purified *Klac*ADA was enzymatically active and capable of utilizing purines. As adenine was the preferred substrate of *Klac*ADA, we determined its kinetic parameters and observed that *Klac*ADA has *K*_M_ 680 ± 4.6 μM and *k*_cat_ 8.1 ± 1.3 s^−1^ with 1.1 × 10^4^ M^−1^s^−1^ efficiency. *Klac*GDA's preferred substrate is guanine. The kinetic parameters of *Klac*GDA for guanine are *K*_M_ 83 ± 2.7 μM and *k*_cat_ 7.3 ± 1.2 s^−1^, respectively, with 0.87 × 10^6^ M^−1^s^−1^ catalytic efficiency (Table 1).

**Table 1:** Kinetic parameters of *Klac*ADA and *Klac*GDA.

**Enzymes**	***K*_M_ (μM)**	***k*_cat_ (s^−1^)**	***k*_cat_/*K*_M_ (M^−1^s^−1^)**
*Klac*ADA	680 ± 4.6	8.1 ± 1.3	1.1 × 10^4^
*Klac*GDA	83 ± 2.7	7.3 ± 1.2	0.87 × 10^5^

### Substrate specificity of *Klac*ADA and *Klac*GDA

Substrate specificity of enzymes was examined by the HPLC-UV method using their respective natural substrates, various purines, and their analogs. As expected, adenine was the preferred substrate for *Klac*ADA, but it is also able to utilize 2,6-diaminopurine, 6-chloropurine, and traces of flavin adenine dinucleotide (FAD), in decreasing order of substrate preference, whereas other compounds showed no detectable activity (Table 2). In the case of *Klac*GDA, guanine is the preferred substrate. *Klac*GDA activity has been examined for other purines (adenine, adenosine, xanthine, hypoxanthine, thioguanine, guanosine, and 8-azaguanine) but it could degrade only 6-thioguanine and 8-azaguanine 13.7 and 23.2%, respectively (Table 3).

**Table 2:** Substrate spectrum of *Klac*ADA.

**Substrates**	**Relative activity (%)**	**Expected product**
Adenine	100%	Hypoxanthine
Xanthine	ND	ND
Deoxyguanosine	ND	ND
FAD	2.0%	Not indentified
Adenosine	ND	ND
Guanosine	ND	ND
Inosine	ND	ND
Xanthosine	ND	ND
2, 6-diaminopurine	86.5%	Guanine
2-chloropurine	ND	Not identified
6-chloropurine	70.7%	Hypoxanthine
Adenosine 5 monophosphate	ND	ND

*ND, Not detected*.

**Table 3:** Substrate spectrum of *Klac*GDA.

**Substrate**	**Relative activity (%)**	**Expected product**
Guanine	100	Xanthine
Xanthine	ND	ND
Adenine	ND	ND
Hypoxanthine	ND	ND
Guanosine	ND	ND
6-thioguanine	13.7	6-thioxanthine
Adenosine	ND	ND
Inosine	ND	ND
8-azaguanine	23.2	8-azaxanthine

*ND, Not detected*.

### Quantification of purine content in beers

Pure purine compounds were used as standards for method optimization. The identification of each purine compound was done according to its relative retention time, and further confirmation was done by LC-ESI-MS analysis. Quantification of the purine content of 16 selected beers that are available in the Indian market revealed that all beers contain different purines like hypoxanthine, adenine, guanosine, guanine, xanthosine, inosine, and uric acid. Overall, the purine content in different beers varied from 91.5 to 339.2 μmol/L, with the brand Beer-KS (<8% of alcohol) being the highest purine-containing beer among 16 different beers (Table 4).

**Table 4:** Purine content in different beers.

**Beer brand**	**Adenine (μMol/L)**	**Guanine (μMol/L)**	**Hypoxanthine (μMol/L)**	**Xanthine (μMol/L)**	**Uric acid (μMol/L)**	**Adenosine (μMol/L)**	**Inosine (μMol/L)**	**Guanosine (μMol/L)**	**Xanthosine (μMol/L)**	**Total Purine content**
Beer-KP	12.05 ± 0.77	–	12.9 ± 0.42	–	–	–	29.3 ± 0.58	–	–	54.3 ± 1.78
Beer-KS	43.5 ± 0.77	69.9 ± 0.07	81.9 ± 0.28	50.5 ± 0.84	–	–	93.5 ± 0.67	–	–	339.45 ± 2.75
Beer-KUL	20.6 ± 0.49	–	39.3 ± 0.5	–	–	–	64.5 ± 1.0	–	4.8 ± 1.0	129.2 ± 3.0
Beer-KUM	29.2 ± 0.78	–	47.0 ± 0.63	–	–	–	86.1 ± 0.30	–	8.2 ± 0.14	171.0 ± 1.83
Beer-BW	26.3 ± 0.56	–	48.5 ± 0.6	–	–	–	–	70.0 ± 0.70	6.0 ± 0.28	151 ± 2.1
Beer-CB	–	11.6 ± 0.35	49.35 ± 0.63	–	–	–	–	56.7 ± 0.77	7.0 ± 1.0	125.0 ± 3.1
Beer-CR	28.5 ± 0.84	–	19.7 ± 0.56	21.9 ± 0.35	–	12.9 ± 0.35	–	7.0 ± 0.42	5.85 ± 0.21	95.9 ± 2.7
Beer-HK	83.2 ± 0.49	–	20.5 ± 0.56	–	–	–	–	–	–	103.7 ± 1.06
Beer-TG	23.7 ± 0.77	34.9 ± 0.21	41.6 ± 0.56	31.9 ± 0.21	–	18.7 ± 0.42	–	–	–	150.9 ± 2.19
Beer-TB	33.1 ± 0.35	59.4 ± 0.49	62 ± 0.42	30.2 ± 0.56	–	–	–	61.9 ± 0.14	8.5 ± 0.49	255.2 ± 2.4
Beer-FL	26.8 ± 0.49	2.75 ± 0.35	81.15 ± 0.77	–	–	–	–	65.8 ± 0.56	–	176.5 ± 2.19
Beer-FG	32 ± 0.42	3.95 ± 0.07	39.15 ± 0.49	2.45 ± 0.21	–	–	–	74.05 ± 0.21	–	151.6 ± 1.4
Beer-MHL	22.65 ± 0.35	–	31.2 ± 0.42	–	–	–	–	58.75 ± 0.78	2.9 ± 0.42	115.5 ± 1.97
Beer-MA	26.0 ± 0.282	–	43.6 ± 0.56	6.3 ± 0.21	–	–	–	15.25 ± 0.5	–	91.2 ± 1.55
Beer-KD	16.6 ± 0.57	2.8 ± 0.42	25.05 ± 0.35	9.7 ± 0.42	–	–	28.1 ± 0.70	40.2 ± 0.35	–	122.5 ± 2.82
Beer-HW	28.6 ± 0.55	5.3 ± 0.14	14.65 ± 0.49	2.09 ± 0.12	8.85 ± 0.21	–	–	40.45 ± 0.91	–	99.94 ± 2.46

### Lowering the purine content in a beer

**Figure 4:**
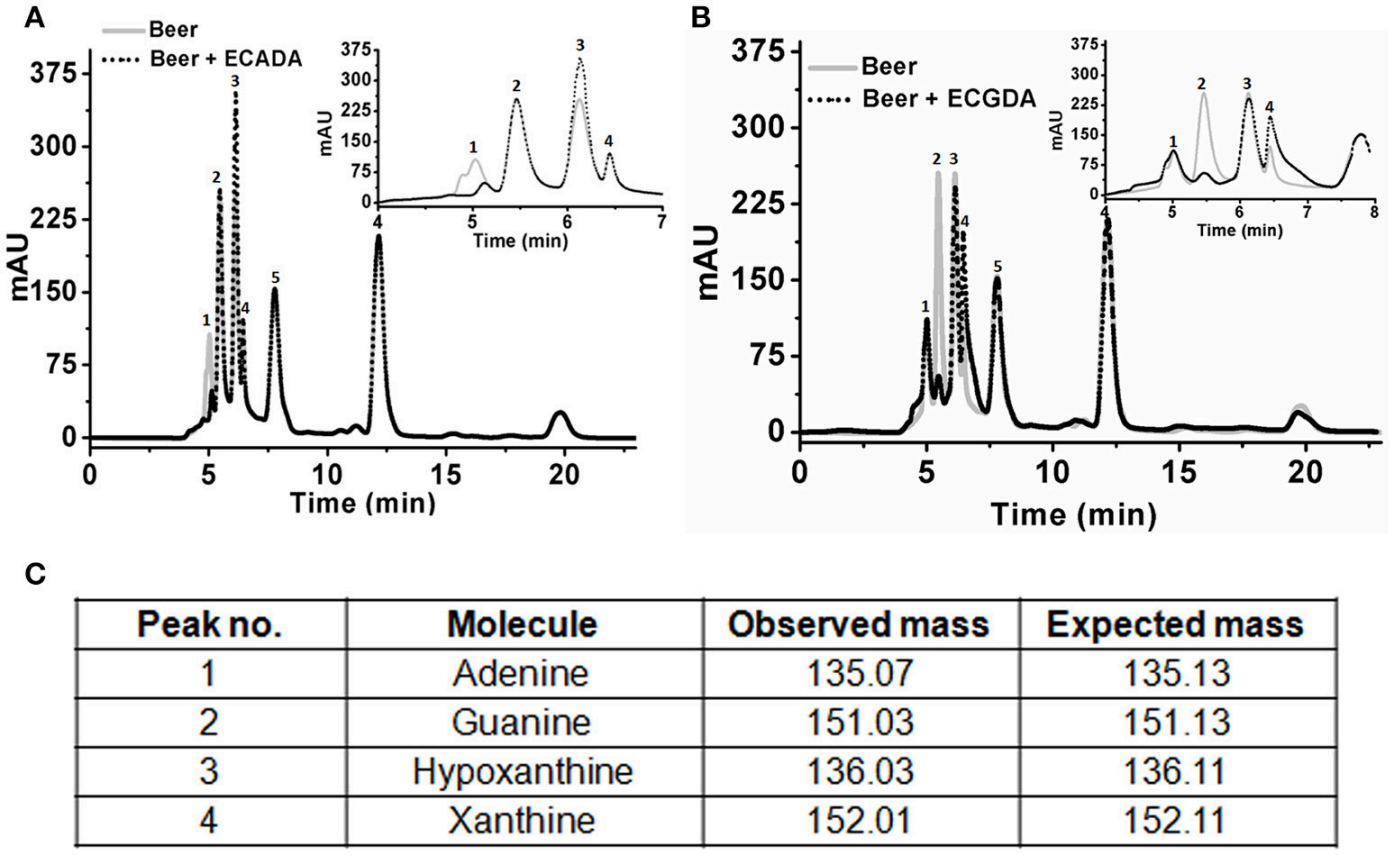
Chromatogram of beer sample before and after the enzyme treatment. **(A)** The chromatogram shows purine content in beer before (dotted black line) and after *Klac*ADA enzyme reaction (solid gray line). **(B)** The chromatogram shows purine content in beer before (dotted back line) and after *Klac*ADA enzyme reaction (solid gray line). Beer sample treated with 0.5 U of *Klac*ADA and *Klac*GDA enzymes. In the panel, A adenine peak (1) was decreased and hypoxanthine peak (3) was increased. In the panel, B guanine (2) peak was decreased and xanthine (3) peak intensity was increased suggesting the conversion of adenine and guanine into hypoxanthine and xanthine, respectively. **(C)** LC-ESI-MS analysis of purines in beer: 1- Adenine, 2- Guanine, 3- Hypoxanthine, 4- Xanthine, expected and observed the molecular mass of purines.

**Figure 5:**
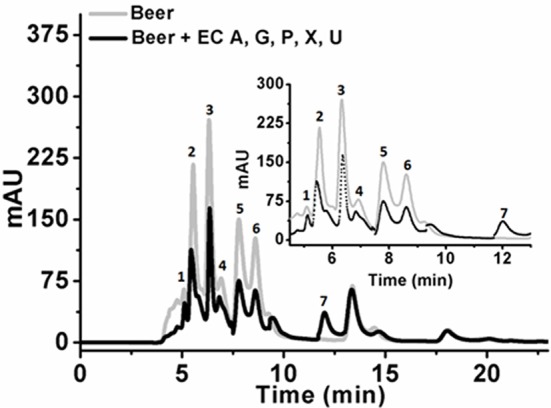
Chromatogram of beer sample before and after the enzyme cocktail treatment. The chromatogram shows purine content in beer before (dotted back line) and after *Klac*ADA, *Klac*GDA, *Klac*PNP, *Klac*XanA, and uricase from *Candida utilis* enzyme reaction (solid gray line). Beer treated with the enzyme cocktail showed a decrease in the adenine, guanine, hypoxanthine, xanthine, and uric acid peaks. EC A (Adenine deaminase), G (Guanine deaminase), P (Purine nucleoside phosphorylase), X (Xanthine dioxygenase) cloned in *E. coli*, and U (commercial uricase).

Initial experiments were performed to examine the ability of *Klac*ADA and *Klac*GDA to reduce the adenine and guanine content in phosphate buffers spiked with these substrates. *Klac*ADA and *Klac*GDA degraded their respective substrates, and it was observed that with a reduction in adenine, there is a corresponding increase in hypoxanthine (Figure S4A), a similar reduction in guanine and a corresponding increase in xanthine (Figure S4B). Similar results were also obtained in beer samples treated with *Klac*ADA and *Klac*GDA (Figure 4). HPLC chromatograms showed that adenine concentration in beer dropped 66–67% and guanine concentration in beer dropped from 68.8 μMol/L to a minimal amount that was difficult to measure (Figure 4). It is evident from analysis that beer samples contain varying concentrations of different purines *viz*. hypoxanthine, inosine, adenine, guanine, and xanthine. To examine the feasibility of reducing overall purine content, a cocktail of enzymes (*Klac*ADA, *Klac*GDA, *Klac*PNP, *Klac*XanA, and commercial uricase from *C. utilis*) was used for treating beer samples. It was observed that the overall purine content of beer decreased. The retention time and LC-ESI-MS analysis revealed that the appeared peak corresponds to allantoin (Figure 5). We have successfully demonstrated that a cocktail of purine degrading enzymes was able to lower the purine content in beer and converted it into a more soluble form that is easy to excrete from the human body.

## Discussion

During the evolutionary process, the loss of human urate oxidase resulted in an increased risk of prevalence to hyperuricemia and gout. Changes in modern lifestyles and diet further enhanced the higher risk of hyperuricemia and gout. Various studies reported that the occurrence of gout has increased worldwide and is dominant in developed countries. Worldwide, cases of gout increased from 0.3 to 6 cases per 1,000 person-years (Kuo et al., 2015). Hyperuricemia and gout are age-associated disorders and are also risk factors for hypertension, metabolic, renal, and cardiovascular diseases. Medications as well as socioeconomic, lifestyle and dietary factors influence the uric acid level in the human body and determine the risk of gout. As 33% of uric acid comes from the diet, consumption of low purine content food and beverages could help to maintain serum uric acid level in humans.

Alcoholic beverages such as beer, spirits, and wine all have different levels of risk of gout. Various studies showed that beer confers the highest risk of gout among all alcoholic beverages (Choi, 2004; Choi and Curhan, 2004, 2008; Choi et al., 2010). One of the reasons for beer's association with gout is the presence of high levels of the nucleoside in beer (Gibson et al., 1984; Puig and Fox, 1984). Some studies reported that two or more servings of beer per day increased the risk of gout by a factor of 2.5 as compared to non-beer drinkers (Choi and Curhan, 2004).

This study reports the characterization of purine degrading enzymes *Klac*ADA and *Klac*GDA, the quantification of purine content in different beers from the Indian market, and examines the application of *Klac*ADA and *Klac*GDA in lowering purine content in beer. Finally, we demonstrated that the application of a cocktail of enzymes (*Klac*ADA, *Klac*GDA, *Klac*PNP, *Klac*XanA with commercial uricase) was successful in lowering purine content in beers.

*Klac*ADA and *Klac*GDA were cloned, overexpressed, and purified from *E. coli* with His-tag. A gel filtration chromatogram revealed that *Klac*ADA and *Klac*GDA exist as a monomer and dimer, respectively, monodispersed in solution. The monomeric form of *Klac*ADA is similar to *A. adeninivorans* ADE (Jankowska et al., 2014). *Klac*ADA showed an optimum pH of 6.0 while maximum ADEs showed an optimum pH range of 6.0–9.0, e.g. *C. fasciculate* (pH 6.0), *A. adeninivorans* (pH 6.5–7.0), *S. cerevisiae* (pH 7.0), *S. pombe* (pH 6.7), and *P. synxantha* (pH 9.0) (Kidder et al., 1977; Jun and Sakai, 1985; Pospísilová et al., 2008). ADEs showed a diverse range of optimum temperatures. For example, *S. cerevisiae, S. pombe, P. synxantha, A. adeninivorans*, and *E. coli* showed optimum temperatures of 30–37, 33, 40–45, 40, and 60°C respectively (Kidder et al., 1977; Jun and Sakai, 1985; Pospísilová et al., 2008; Jankowska et al., 2014). It was found that *Klac*ADA's optimum temperature is 30°C, which is nearer to *S. cerevisiae* and *S. pombe*. Some studies reported that ADEs have ~30% activity in the 5.0–8.0 pH range (Kidder et al., 1977; Jun and Sakai, 1985). *A. adeninivorans* ADE is stable at 30°C up to 24 h in the HEPES buffer, ranging from 7.5 to 8.5 (Jankowska et al., 2014). *Klac*ADA's pH stability was tested in the universal buffer and found that it can work at a wide pH range of 5.0–9.0. *C. fasciculate* ADA is a thermostable enzyme and retained 90% activity at up to 55°C for 1 h (Kidder et al., 1977). ADE of *C. utilis* was an unstable protein and lost 50% of activity within 10 min at 37°C. *A. adeninivorans* ADE retained 70% of activity after 1 h at 40°C (Jankowska et al., 2014). *Klac*ADA is moderately thermostable and able to work at 5–40°C with >70% activity and able to work across wide pH range.

Allam et al. (1981) reported that ADE has only a natural substrate, adenine, but other studies showed that ADE can also hydrolyze 6-halogenpurines (Abbondandolo et al., 1971; Jun and Sakai, 1985). It was observed that *Klac*ADA is able to consume adenine, 2,6-diaminopurine, 6-chloropurine, and 8-azaguanine. It was described that adenine deaminase can consume various structurally related cyclic compounds as substrates (Hartenstein and Fridovich, 1967). The kinetic parameters of *Klac*ADA for adenine are *K*_M_ 680 ± 4.6 μM, *k*_cat_ 8.1 ± 1.3 s^−1^, with a catalytic efficiency of 1.1 × 10^4^ M^−1^s^−1^. The *K*_M_ value of ADEs has been reported from 40 different clones (wild-type and mutants), which lay in the range of 0.09–2.2 mM (Brenda Database). *Klac*ADA lies in the mentioned range and is more catalytically efficient in comparison to many ADEs, such as *E. coli, Burkholderia* sp., *P. aeruginosa* (Goble et al., 2011; Kamat et al., 2011).

The optimum pH of GDA ranges from 5.5 to 9.0 in the different organisms and it was found that *Klac*GDA's optimum pH was 7.0, which is comparable to the pH of humans and *Camellia sinesis*. GDA is a moderately thermostable enzyme that can work actively up to 55°C (Trautwein-Schult et al., 2014). It was observed that *Klac*GDA's optimum temperature was 25°C, which is comparable with the optimum temperature of *O. cuniculus* (Ujjinamatada et al., 2006). *Klac*GDA is a pH-stable protein and retained ~50% activity at pH 5.0–9.0. The pH stability data suggest that *Klac*GDA has a broad range of pH stability in comparison to *A. adeninivorans* GDA (Trautwein-Schult et al., 2014). Various studies reported that GDA is a thermostable enzyme; *Rattus norvegicus* GDA retained 67% at 70°C (Kim and Kimm, 1982) and Human liver GDA is active up to 55°C (Kimm and Lee, 1985). In our study, it was found that *Klac*GDA has >40% activity at a range of 5–40°C, which is comparable to human liver GDA (Gupta and Glantz, 1985). Guanine and ammeline are natural substrates for GDA. Besides these two substrates, GDA is also able to catalyze other analogs (6-thioguanine and 8-azaguanine). We found the *Klac*GDA consumes guanine with high efficiency but is unable to consume other purines except for the 6-thioguanine and 8-azaguanine. *Klac*GDA has higher *K*_M_ as compared to the reported *K*_M_ of human liver, *A. adeninivorans, C. sinensis*, and *R. norvegicus* (Kimm and Lee, 1985; Negishi et al., 1994; Trautwein-Schult et al., 2014).

From the literature survey, we could not find any report on purine content analyses of beer samples available from the Indian market. Purine content in beer may depend on the source of raw material used, strain selection, and downstream processing. So, before treating with enzymes, a quantification of purine content was done in different beers available in the Indian market. It was observed that hypoxanthine, adenine, guanine, guanosine, inosine, and uric acid were present in different beers and hypoxanthine was high in each beer in comparison to the purine content in beer from the UK and Japan. Hypoxanthine is the most harmful purine in the context of gout.

The beer samples treated with *Klac*ADA and *Klac*GDA showed a reduction in the amount of adenine and guanine, respectively. Furthermore, the beer was treated with an enzyme cocktail and it was observed that the overall purine content of beer was reduced significantly. This is a preliminary study which demonstrated the application of an enzymatic approach to low purine content beverage production. Further studies are required to develop a method for industrial applications of this approach. A similar approach could also be developed for lowering purine content in other kinds of foods. Currently, our group is exploring strategies to make low purine content food affordable. We believe, using yeast strain harboring engineered enzymes like *Klac*ADA, *Klac*GDA, *Klac*PNP, and *Klac*XanA for the production of beer could be an attractive and cost-effective approach for creating low purine content beverages and foods. The application of this approach may be beneficial to hyperuricemic and gouty patients in maintaining healthy life styles.

## Author contributions

GP conceived and coordinated the study. DM designed and carried out experiments. DM and GP analyzed the data and wrote the paper. All authors reviewed results and approved the final version of the manuscript.

### Conflict of interest statement

The authors declare that the research was conducted in the absence of any commercial or financial relationships that could be construed as a potential conflict of interest.

## Supplementary material

Figure S1 Amino acid sequence alignment of *Klac*ADA with the adenine deaminases of *S. cerevisiae, K. marxianus*, and adenosine deaminases (ADAs) of Human, *E. coli, Bos Taurus*, and *Mus musculus*. Highly Conserved regions are highlighted in red boxes, conservative substitutions are also boxed. The amino acid similarity figure was generated by using the ESPript 3.

Figure S2 Amino acid sequence alignment of *Klac*GDA with the guanine deaminases of Human, *S. cerevisiae, E. coli*, and *K. marxianus*. Highly Conserved regions are highlighted in red boxes, conservative substitutions are also boxed. The amino acid similarity figure generated by using the ESPript 3.

Figure S3 MALDI-TOF profile of *Klac*ADA and *Klac*GDA. **(A)** MALDI-TOF analysis of *Klac*ADA revealed the monomeric form of it. **(B)** MALDI-TOF profile of *Klac*GDA revealed the monomeric and dimeric form of it.

Figure S4 HPLC chromatograms of *Klac*ADA and *Klac*GDA with the standards. **(A)** Adenine treated with the *Klac*ADA (solid gray line), HPLC chromatograms showed the reduction in adenine peak (solid black line) and corresponding elevation in hypoxanthine peak (dotted gray line). **(B)** Guanine treated with the *Klac*GDA (solid gray line), HPLC chromatograms showed the reduction in guanine peak (solid black line) and corresponding elevation in xanthine peak (dotted gray line).
