# Supplementary figures and images for: Biochemical Characterization of Kluyveromyces lactis Adenine Deaminase and Guanine Deaminase and Their Potential Application in Lowering Purine Content in Beer
Source: Front Bioeng Biotechnol. 2018 Nov 29;6:180. doi: 10.3389/fbioe.2018.00180 (PMC6281700; doi:10.3389/fbioe.2018.00180)

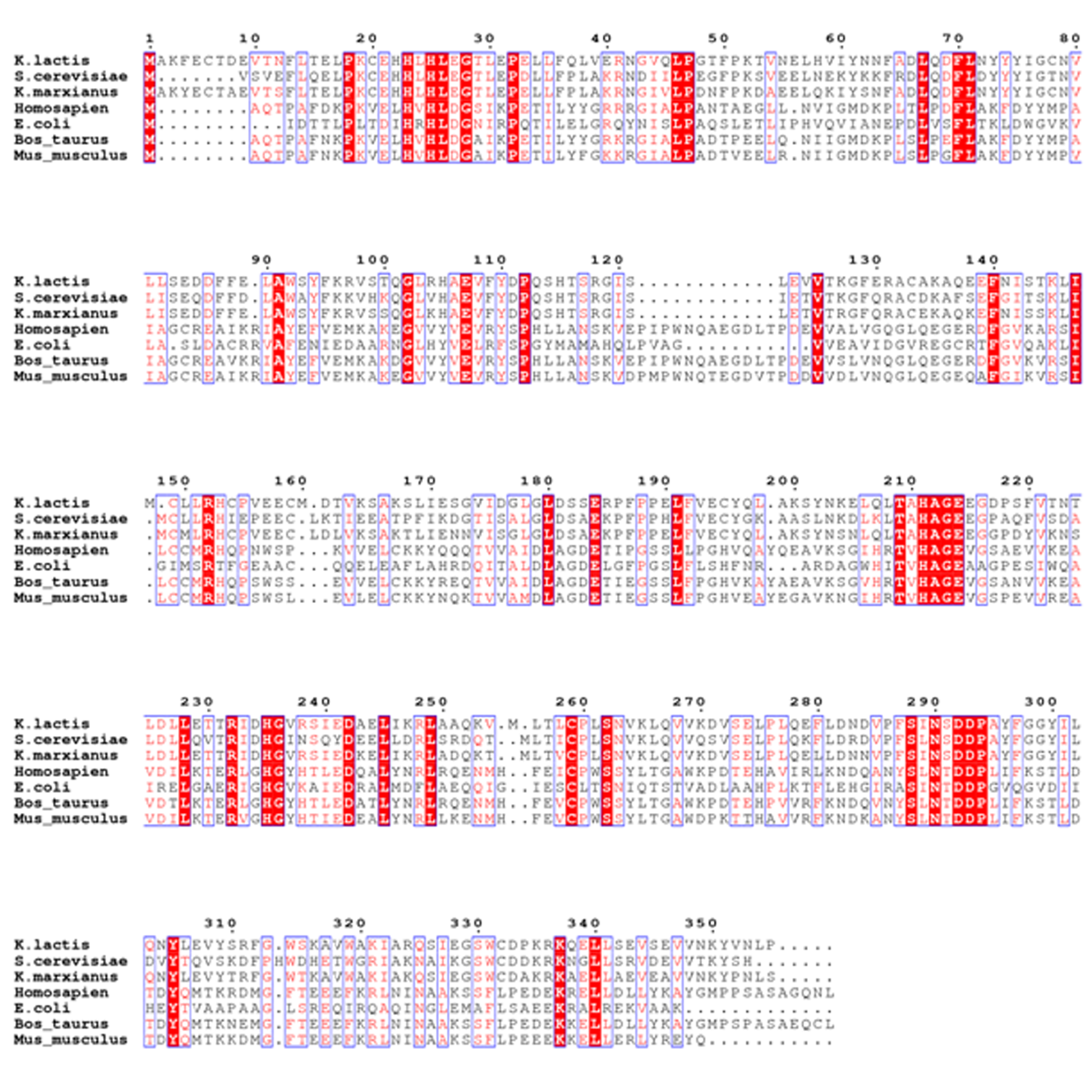

Supplement: Figure S1 — Amino acid sequence alignment of KlacADA with the adenine deaminases of S. cerevisiae, K. marxianus, and adenosine deaminases (ADAs) of Human, E. coli, Bos Taurus, and Mus musculus. Highly Conserved regions are highlighted in red boxes, conservative substitutions are also boxed. The amino acid similarity figure was generated by using the ESPript 3. [file Image_1.TIF]

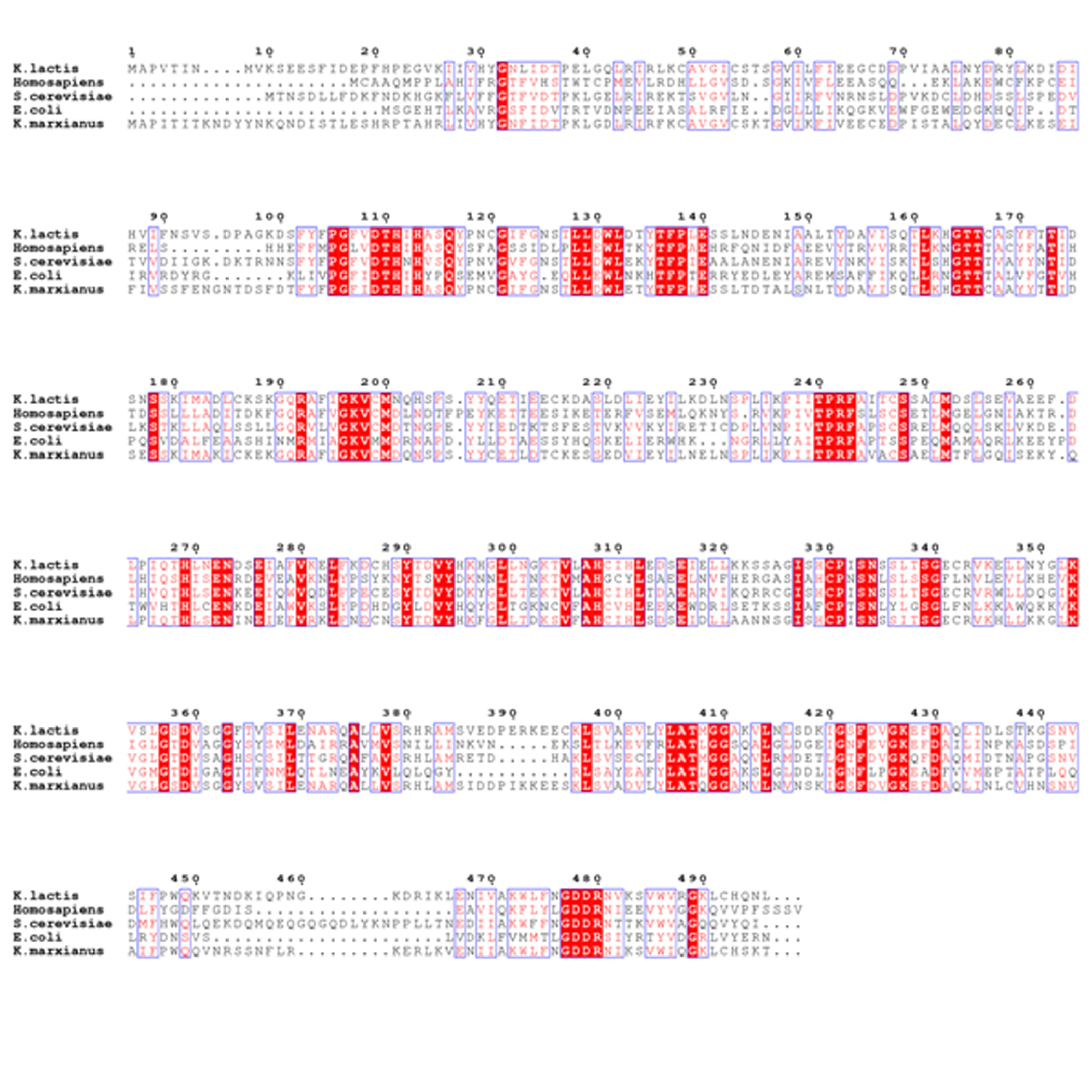

Supplement: Figure S2 — Amino acid sequence alignment of KlacGDA with the guanine deaminases of Human, S. cerevisiae, E. coli, and K. marxianus. Highly Conserved regions are highlighted in red boxes, conservative substitutions are also boxed. The amino acid similarity figure generated by using the ESPript 3. [file Image_2.TIF]

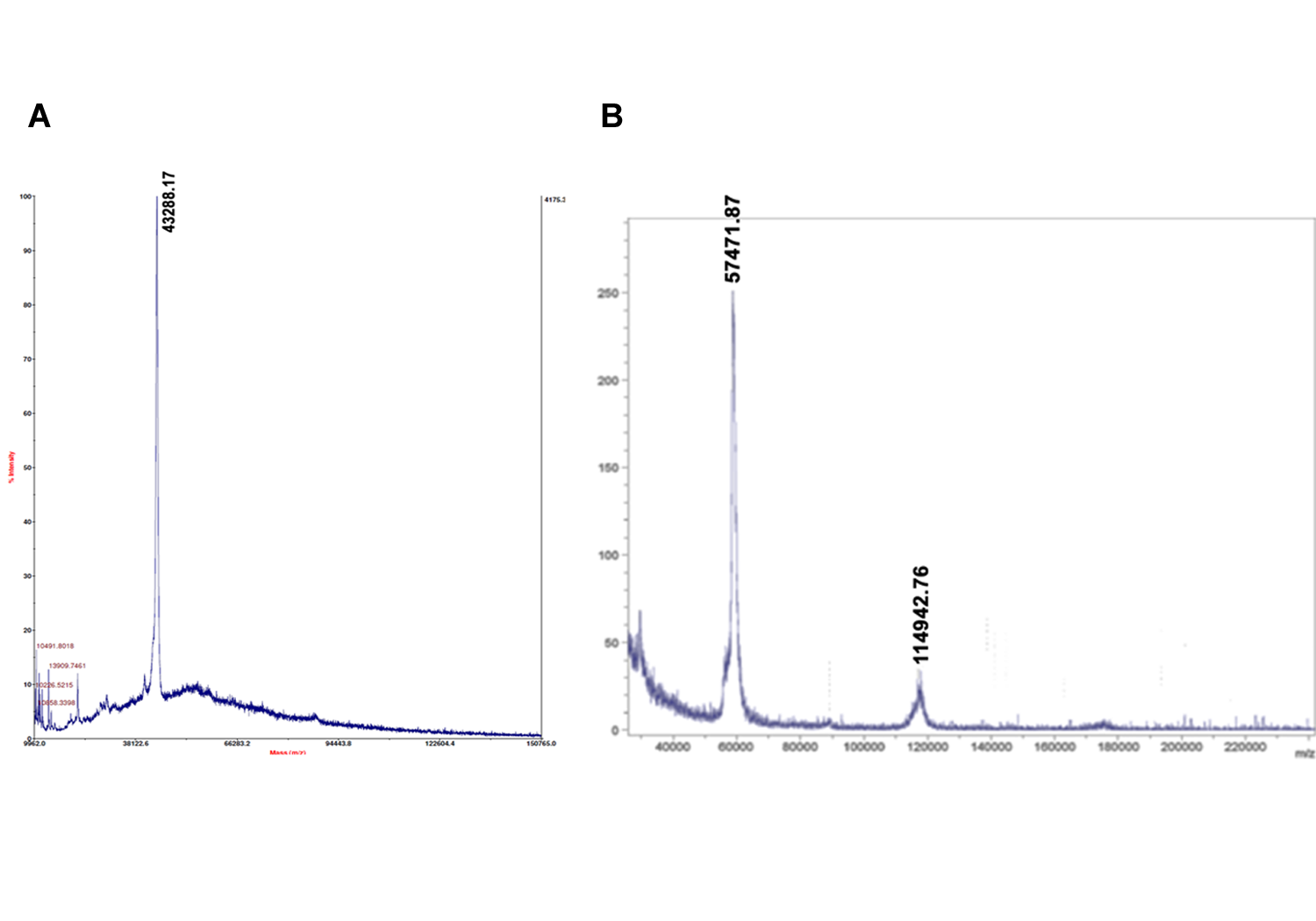

Supplement: Figure S3 — MALDI-TOF profile of KlacADA and KlacGDA. (A) MALDI-TOF analysis of KlacADA revealed the monomeric form of it. (B) MALDI-TOF profile of KlacGDA revealed the monomeric and dimeric form of it. [file Image_3.TIF]

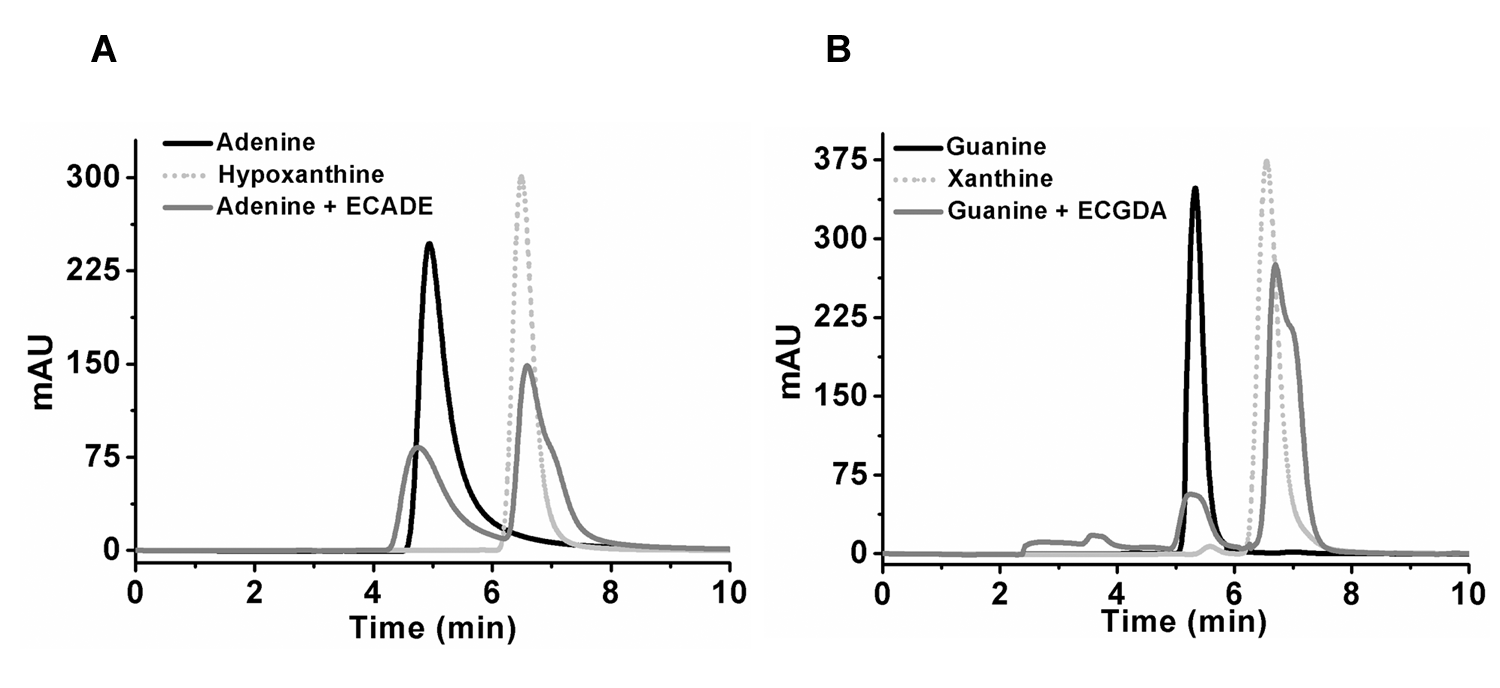

Supplement: Figure S4 — HPLC chromatograms of KlacADA and KlacGDA with the standards. (A) Adenine treated with the KlacADA (solid gray line), HPLC chromatograms showed the reduction in adenine peak (solid black line) and corresponding elevation in hypoxanthine peak (dotted gray line). (B) Guanine treated with the KlacGDA (solid gray line), HPLC chromatograms showed the reduction in guanine peak (solid black line) and corresponding elevation in xanthine peak (dotted gray line). [file Image_4.TIF]
